# Prevalence of ATM Sequence Variants in Northern Plains American Indian Cancer Patients

**DOI:** 10.3389/fonc.2013.00318

**Published:** 2013-12-30

**Authors:** Daniel G. Petereit, L. Jennifer Hahn, Shalini Kanekar, Amy Boylan, Søren M. Bentzen, Mark Ritter, Amy R. Moser

**Affiliations:** ^1^Rapid City Regional Hospital, John T. Vucurevich Cancer Care Institute, Rapid City, SD, USA; ^2^Department of Human Oncology, University of Wisconsin School of Medicine and Public Health, Madison, WI, USA

**Keywords:** radiogenomics, ATM variants, American Indians, radiosensitivity

## Abstract

**Purpose**: To identify sequence variants of the ataxia telangiectasia mutated (*ATM*) gene and establish their prevalence rate among American Indian (AI) as compared with non-AI cancer patients.

**Materials and Methods**: DNA was isolated from blood samples collected from 100 AI and 100 non-AI cancer patients undergoing radiation therapy, and a blinded assessment of the ATM sequence was conducted. Quantitative PCR assessment of copy number for each exon was also performed. The main outcome measure was the prevalence of ATM variants in the two patient populations.

**Results**: No statistically significant differences for total prevalence of ATM variants among AI and non-AI patients were found. Of the 25 variants identified, 5 variants had a prevalence of >2%, of which 4 occurred at a rate of >5% in one or both groups. The prevalence of these four variants could meaningfully be compared between the two groups. The only statistically significant difference among the groups was the c.4138C > T variant which is predicted not to affect protein function, seen in 8% of AI versus 0% of non-AI patients (*P* = 0.007). No exonic copy number changes were found in these patients.

**Conclusion**: This study is the first to determine the prevalence of ATM variants in AIs.

## Introduction

Radiation has a pivotal role in the management of human cancer with approximately 50% of all cancer patients receiving it at some point during their course of treatment. While radiation therapy is often successful in the local eradication of tumor cells, late normal tissue effects may significantly reduce organ function and health-related quality of life in a proportion of long term cancer survivors (1). Individual variability in normal tissue response to fractionated radiotherapy has been observed in both clinical and laboratory studies. The risk of radiation induced toxicities is affected by treatment related factors such as total radiation dose, dose per fraction, volume of normal tissue irradiated, and other patient related factors (2, 3). These factors alone are not sufficient to account for the variability seen among patients, however. Hence, identifying new genetic biomarkers having potential relevance to toxicities and incorporating these into traditionally used assessment tools will help personalize treatment plans and potentially increase efficacy and reduce toxicities.

The induction and processing of radiation damage to normal cells and tissues involve multiple molecular pathways from DNA damage repair to wound healing responses and tissue remodeling (4). Genetic variability in genes that regulate these processes can either play a protective role or render an individual susceptible to radiation induced cell damage. Ataxia telangiectasia mutated (ATM) is a serine threonine kinase that phosphorylates several substrates involved in diverse functions such as repair of double stranded breaks in DNA, cell cycle regulation, radiosensitivity, and oxidative stress. A-T is an autosomal recessive disorder and is a result of deleterious mutation in the ATM gene (5). Typical manifestations are neurological deterioration and ocular telangiectasia and increased susceptibility to radiation toxicities (6).

Skin fibroblasts from A-T patients show a threefold increase in radiosensitivity when irradiated *in vitro* compared to normal human fibroblasts (7). Although A-T homozygotes are rare, A-T heterozygosity is estimated to occur in about 1–2% of the Caucasian population in the US (8). Heterozygosity for mutations shown to result in AT may confer a greater risk of developing cancer and increased radiosensitivity to carriers (9).

American Indians (AIs) appear more sensitive to fractionated radiotherapy as reported by our group. This was evident by AIs developing higher rates of G3 skin and mucosal reactions as compared to non-AI patients (10). The ATM gene has not been studied in Northern Plains AIs, a population that exhibits disproportionate burden of cancer mortality. We hypothesized that a significant proportion of AI that manifest toxicities in response to radiation therapy carried deleterious variants in the ATM gene. In this study we aimed to determine the prevalence of ATM variants among AI cancer patients and compare this to non-AI cancer patients. The ATM study is one of many conducted by our group known as Walking Forward which has been in existence since 2002. This NCI funded program addresses cancer disparities through patient navigation, enrollment to clinical trials, and determinants of cancer screening and delays to cancer diagnosis and treatment (11).

## Materials and Methods

### Patients

Peripheral blood was obtained from 202 patients (101 AIs and 101 non-AIs) seen during a routine treatment visit or follow-up. Patients were staged according to American Joint Committee on Cancer standards. 10.5% of patients were treated with palliative intent and 89.5% with curative intent. The study was approved by the Rapid City Regional Hospital’s Institutional Review Board and by all Tribal entities that were a part of this study. All patients who participated were consented.

### DNA extraction and sequencing

Genomic DNA was extracted from peripheral blood lymphocytes via the PAXgene Blood DNA Kit (Qiagen) and dissolved into 600 μl volumes of Buffer BG4 (Qiagen). The concentration of the dissolved gDNA was determined using a SpectraMax 190 Microplate Reader (Molecular Devices) or Nanodrop 1000 spectrophotometer (Thermo Scientific).

### ATM exon characterization

Coding exons 4 through 65 and flanking intronic regions were amplified individually, except for exons 4 and 5, 23 and 24, and 43 and 44 which were amplified as single products. Exon numbering is based on transcript NM_000051.3 as described elsewhere (12). We used a set of primers as previously published (13) with a few alterations. Amplification was done with the MasterTaq kit (5′) in 20 μl volumes consisting of 1× TaqMaster solution, 1.5 mM Mg^2+^, 0.2 mM mixed dNTP’s, 0.5 U/μl taq, 8 pmols of sense primer, 8 pmols of anti-sense primer, and ~125 ng gDNA in reagent grade water. Sequencing was done with the Big Dye v3.1 solution in 10 μl volumes using 4 pmol of one primer, 0.25 μl Big Dye v3.1, 3.75 μl 2.5× buffer (19 mM Tris-HCl, 180 mM Tris, 5 mM MgCl_2_, pH 9.0 ± 0.5), ~1 ng DNA per 100bp of amplicon length and reagent grade water.

Multiplex PCR was done to test the copy number of each exon. An unrelated non-polymorphic marker on chromosome 7 was included in each mix to serve as a non-*ATM* control peak. About ~125 ng gDNA was amplified with 5 μl 2× Master Mix from the Multiplex PCR kit (Qiagen), 2 μl of a 0.5:50 μM mix of sense to anti-sense primers, and 1 μl each of 7 μM FAM- or HEX-labeled additional universal sense primers. The sense primers for each exon were designed to bind one of the universal sense primers. Amplification products were diluted 1:5 with water. GeneFlo 625 DNA Markers (ChimerX) were diluted 0.3 μl in 15 μl distilled water per sample. Then 1 μl of diluted amplicons was mixed with 15.3 μl diluted markers, denatured at 95°C, and immediately cooled. The products were analyzed with an ABI 3730xl DNA analyzer.

### Variation discovery and characterization

Traces were checked for quality, processed into a multi-alignment assembly for each exon, and scanned for variations using visual inspection, SequenceScanner v1.0 (Applied Biosystems), and the Staden Package 1.6.0 for Mac and 2002 for Windows (14). Potential variants were confirmed based on sequencing in the opposite direction and/or a second or third sequencing run. Variants were named according to the recommendations of the Human Genome Variation Society. PolyPhen-2 (15), a software tool that predicts the likely impact of an amino acid substitution on the function of a human protein, was run on each variant.

Fragment files from the exon copy number tests were downloaded and analyzed without additional manipulation in Peak Scanner v1.0 (Applied Biosystems).

### Statistics

Fisher’s Exact Test was used to test for an association between ethnicity and prevalence of a specific genotype. Analyses were performed using IBM SPSS version 20.0.0. Statistical significant associations were defined as those where the two-sided *P*-value was lower than or equal to 0.05. Prevalence estimates are specified in percent together with their exact 95% confidence limits.

### Obtaining consent

While there were initial concerns that this study could not be opened, or accrued to, due to concerns of genetic testing in a disparate population, IRB approval was rapidly obtained with only a few patients refusing participation. Patients were eager to enroll as they believed it could help the next generation of cancer patients.

## Results

A total of 25 variants were identified in this patient population; 6 would be predicted to be silent, and 19 would be predicted to result in change in the protein sequence (Table 1). None would be predicted to truncate the protein. Of those missense variants, 14 were predicted to be possibly or probably damaging to protein function. None of the variants identified in this study have been associated with the development of A-T. One of the variants (7228 T > C) found in one non-AI patient, was not previously registered in the NCBI dSNP database. No statistically significant difference for total prevalence of variants was found among AI (40%) and non-AI (48%) patients (*P* = 0.32). Nineteen of the variants were found in the non-AI patients and 12 in the AI patients. Five variants had a prevalence of ≥2%, of which four occurred at a rate of >5% in one or both groups (Table 2). The prevalence of these could meaningfully be compared statistically in the two groups. The only statistically significant difference among the groups was the c.4138C > T variant seen in 8% of AI versus 0% of non-AI patients (*P* = 0.007). However, the PolyPhen-2 software tool predicts that this variant (His1380Tyr) will not affect protein function. Also another variant, variant c.5557G > A, had a prevalence of 25% in non-AI versus 14% in AI patients (*P* = 0.07) but is predicted not to affect protein function. Three patients are homozygous for this variant, all in the non-AI group. There was one additional non-AI patient who is homozygous for the c.378C > T variant which is also predicted to be benign. The prevalence of those variants predicted to result in potentially deleterious missense mutations was 7% among non-AI and 7% among AI patients.

**Table 1:** **List of exonic variants**.

cDNA-level variation name (based on LRG_135)	rs No.	Exon	Theoretical protein-level variation name	PolyPhen-2^a^ results (humdiv-trained)	*n* Total patients	*n* Caucasian patients	*n* Native American patients
**Non-synonymous variants**
c.[94C > T];[ =]	rs148061139	05	p.[(Arg32Cys)];[( =)]	Probably damaging	1	1	0
c.[146C > G];[ =]	rs1800054	05	p.[(Ser49Cys)];[( =)]	Probably damaging	1	0	1
c.[202A > G];[ =]	rs35389822	06	p.[(Ile68Val)];[( =)]	Benign	1	1	0
c.[378T > A];[378T > A]	rs2234997	07	p.[(Asp126Glu)];[(Asp126Glu)]	Benign	1	1	0
c.[998C > T];[ =]	rs28904919	10	p.[(Ser333Phe)];[( =)]	Possibly damaging	1	1	0
c.[1229T > C];[ =]	rs56128736	11	p.[(Val410Ala)];[( =)]	Possibly damaging	2	1	1
c.[1744T > C];[ =]	rs2235006	13	p.[(Phe582Leu)];[( =)]	Benign	1	0	1
c.[1960C > A];[ =]^b^	–	15	p.[(Gln654Lys)];[( =)]	Benign	1	1	0
c.[2119T > C];[ =]	rs4986761	15	p.[(Ser707Pro)];[( =)]	Benign	2	2	0
c.[2149C > T];[ =]	rs147515380	16	p.[(Arg717Trp)];[( =)]	Probably damaging	1	1	0
c.[2572T > C];[ =]	rs1800056	19	p.[(Phe858Leu)];[( =)]	Possibly damaging	1	0	1
c.[3161C > G];[ =]	rs1800057	24	p.[(Pro1054Arg)];[( =)]	Probably damaging	4	1	3
c.[3383A > G];[ =]	rs56398245	25	p.[(Gln1128Arg)];[( =)]	Possibly damaging	1	0	1
c.[4138C > T];[ =]	rs3092856	30	p.[(His1380Tyr)];[( =)]	Benign	8	0	8
c.[4258C > T];[ =]	rs1800058	31	p.[(Leu1420Phe)];[( =)]	Benign	9	7	2
c.[5071A > C];[ =]	rs1800059	36	p.[(Ser1691Arg)];[( =)]	Benign	1	1	0
c.[5557G > A];[ =]	rs1801516	39	p.[(Asp1853Asn)];[( =)]	Benign	36	22	14
c.[5557G > A];[5557G > A]	rs1801516	39	p.[(Asp1853Asn)];[(Asp1853Asn)]	Benign	3	3	0
c.[5558A > T];[ =]^c^	rs1801673	39	p.[(Asp1853Val)];[( =)]	Possibly damaging	1	1	0
c.[7228T > C];[ =]^d^	rs193302874	51	p.[(Phe2410Leu)];[( =)]	Probably damaging	1	1	0
				**Non-synonymous variant subtotals**	**77**	**45**	**32**
**Synonymous variants**
c.[162T > C];[ =]	rs3218690	05	(p. =) Tyr54	N/A	1	0	1
c.[657T > C];[ =]	rs2235003	08	(p. =) Cys219	N/A	1	1	0
c.[735C > T];[ =]	rs3218674	09	(p. =) Val245	N/A	2	1	1
c.[4578C > T];[ =]	rs1800889	32	(p. =) Pro1526	N/A	23	11	12
c.[5793T > C];[ =]	rs3092910	41	(p. =) Ala1931	N/A	2	2	0
c.[8592C > T];[ =]	rs56025670	61	(p. =) Tyr2864	N/A	1	1	0
				**Synonymous variant subtotals**	**30**	**16**	**14**
Nomenclature style is taken from the Human Genome Variation Society recommendations, found at http://www.hgvs.org/mutnomen/				**Variant totals for all**	**107**	**61**	**46**

^a^PolyPhen-2 v2.2.2, updated February 15, 2012. Available from: http://genetics.bwh.harvard.edu/pph2/index.shtml

*^b^While not in dbSNP, this variant was noted in Ref. (16)*.

*^c^There is one Caucasian patient who had an indeterminate genotype in this codon. The patient is represented in each heterozygous category independently in this table. c.[5557G > A (;) 5558A > T] p.[(Asp1853?)];[(?)]. Amino acid change possibilities: AAT, asparagine (polar, neutral, benign); ATT, isoleucine (non-polar, neutral, possibly damaging); GAT, no change on one allele; GTT, valine (non-polar, neutral, possibly damaging)*.

*^d^This variant was not in any publications or in dbSNP prior to our submission*.

**Table 2:** **Prevalence (95% confidence limits) of most common ATM variants**.

SNP	American Indians	Non-American Indians	*P*-value*	PolyPhen
c.5557G >A	14% (8%, 22%)	25% (17%, 35%)^a^	0.07	Probably benign
c.4578C >T	12% (6%, 20%)	11% (6%, 19%)	1.0	NA^b^
c.4258C >T	2% (0.2%, 7%)	7% (3%, 14%)	0.17	Benign
c.4138C >T	8% (4%, 15%)	0% (0%, 4%)	0.007	Benign
c.3161C >G	3% (0.6%, 9%)	1% (0.03%, 4%)	0.61	Probably damaging
Any variant	40% (30%, 50%)	48% (38%, 58%)	0.32	–

*P-value for the null hypothesis of equal prevalence in American Indians and non-American Indians patients.

*^a^Three homozygous cases*.

*^b^Synonymous change*.

Sixteen patients had more than one variant, in four cases this involved the c.4578C > T and c.5557G > A pair of variants (Table 3). Two patients had three variants. We were unable to determine the haplotype for any of the patients with multiple variants.

**Table 3:** **Summary of ATM variants**.

Patients with multiple variant combinations			Number of patients with combination
c.998C >T	c.5557G >A		1
c.1229T>C	c.5557G>A		1
c.4578C>T	c.5557G>A		4
c.378T>A	c.657T>C		1
c.2119T >C	c.2149C >T	c.4578C >T	1
c.4258C>T	c.5071A>C		1
c.4258C>T	c.4578C>T		1
c.735C>T	c.4258C>T		1
c.5557G>A	c.5558A>T		1
c.2572T>C	c.3161C>G		1
c.202A>G	c.3161C>G	c.5557G >A	1
c.146C>G	c.5557G>A		1
c.4138C>T	c.4578C>T		1

We performed an analysis for exonic copy number and found no variations in exonic copy number within this population.

## Discussion

As part of the original research design, the ATM gene was investigated as there was concern that the AI patient population was more sensitive to the effects of therapeutic radiation. Fear of this treatment related side-effect could potentially contribute to the commonly observed treatment delays observed in this patient population. A total of 101 AI and 101 non-AI participated in the ATM study (17). This study provided new information on the ethnic distribution of ATM variants in a regional US population, and is the first to determine the prevalence of ATM variants in AI cancer patients.

Our results indicate that the variants found in the AI patient population, with the exception of the 4138C > T variant, are also found in the non-AI population. In fact, there were more variants identified in the non-AI population. The most prevalent variant found in both populations was the 5557 G > A variant – resulting in the replacement of aspartic acid with asparagine, a substitution that the PolyPhen-2 tool predicts to be benign. At the time of writing, there are at least 11 published studies of the possible link between this particular variant and normal tissue toxicity after radiation therapy. The outcomes of these studies are inconsistent, ranging from studies finding an increased risk of toxicity among carriers of the minor allele to a decreased risk, and with several studies finding no significant link. A large individual patient level meta-analysis of all the available data is being conducted by the international Radiogenomics Consortium (RGC), see below.

The c.5557G > A SNP has also been intensively studied with respect to possible association with breast cancer susceptibility. Recent meta-analyses found no evidence of such an association (18), although interestingly in the present context, in a sub-group analysis stratifying for ethnicity AIs did show an increased odds ratio of 2.19 (with 95% CI 1.38–3.47). However, further studies are needed to confirm this finding.

A recent attempt at validating a large number of SNP’s that have previously been reported by various groups to be associated with toxicity after radiation therapy showed that not a single one of these could be independently validated, in other words the published literature is dominated by false-positive associations (19). In an attempt to synthesize information from multiple studies and to push the number of cases in future studies into the range of 2,000–10,000 cases, an international RGC was formed in November 2009 (20). The RGC has more than 110 members mainly from Asia, Europe, and North America with expertise in radiation biology, radiation oncology, epidemiology, genetics, radiation physics, statistics, or bioinformatics. The data from the present study are also included in the RGC virtual databank. Relatively small studies like the present one are of great potential value as haplotypes will tend to differ between various ethnic groups. One could envision a research strategy where the predictive strength of various variants was established in the larger cohorts, potentially using genome wide association methodologies (discovery phase), validated in the same population whereafter the most promising among the validated variants would subsequently be tested in populations such as the AI. It is also possible that rare variants could be relatively more common in some populations due to founder mutations. However, in the present study there was no evidence of this, with the possible exception of the c.4138C > T SNP among the AI patients.

It was originally planned to correlate the variant data to patient toxicity data with the hopes of finding a predictive assay. However, the analysis was not performed because of the heterogeneity in disease sites, and the fact that the distribution among sites were different in the AI and non-AI groups. It is more realistic that discovery of variants linked with toxicity will come from much larger populations, and once strong candidate variants have been identified the prevalence of these among the AI patients can be established.

## Conclusion

There is no support in our study for the hypothesis that a difference in prevalence or type of ATM variants explains the different spectrum of side-effects seen after radiotherapy in AI versus non-AI patients. Correlation to variant and toxicities was not performed as the study was underpowered. The successful completion of this study is indicative of the trust we have garnered with tribal entities throughout Western SD, and has positioned us to now become part of the worldwide RGC, where we will help to potentially solve the mystery of finding a predictor of increased radiosensitivity.

## Conflict of Interest Statement

The authors declare that the research was conducted in the absence of any commercial or financial relationships that could be construed as a potential conflict of interest.
